# Production of mouse androgenetic embryos using spindle perturbation

**DOI:** 10.1038/s41598-020-63010-x

**Published:** 2020-04-16

**Authors:** Takaya Totsuka, Miho Ohsugi

**Affiliations:** 1 Department of Biological Sciences, Graduate School of Science, The University of Tokyo, Hongo 7-3-1, Bunkyo-ku, Tokyo 113-0033 Japan; 2 Department of Life Sciences, Graduate School of Arts and Sciences, The University of Tokyo, Komaba 3-8-1, Meguro-ku, Tokyo 153-8902 Japan

**Keywords:** Biological techniques, Developmental biology, Embryogenesis, Cell biology, Cell division

## Abstract

To study the functional differences between maternal and paternal genomes in mammalian development, embryos with only one parental genome are often used. Androgenetic embryos are produced by the removal of maternal chromosomes before or after fertilization by techniques that require specialized skills and are associated with high risk of cellular damage. Here, we developed a novel method for producing androgenetic mouse embryos without the invasive enucleation process. We found that during *in vitro* fertilization in the presence of low-dose nocodazole, a microtubule destabilizing drug, whole oocyte chromosomes were extruded into the second polar body resulting in the production of androgenetic embryos. We further demonstrated that low-dose nocodazole decreased the spindle size and prevented chromosome segregation but did not compromise oocyte meiotic resumption. This led to the formation of a protrusion around the chromosomes, accumulation of protein regulator of cytokinesis 1 (PRC1) to the microtubules around the chromosomes, and assembly of a contractile ring at the neck region of the protrusion. Our method uses the intrinsic cytokinetic mechanism to exclude maternal chromatin from zygotes and may be applicable to other mammals.

## Introduction

The cell cycle in unfertilized vertebrate oocytes is arrested at the metaphase of the second meiosis (Meta-II). Sperm-oocyte fusion triggers the meiotic resumption and onset of the anaphase-II (Ana-II), leading to sister chromatid segregation toward the spindle poles. As a result of highly asymmetric cell division, one set of sister chromatids is extruded to the small second polar body, and the other forms a maternal pronucleus (PN) in the large zygote^1^. Polar body formation is typically a process of cytokinesis with an oocyte-specific feature. The Meta-II spindle is located close to the cortex, and aligned chromosomes induce the formation of a thick cortical actin meshwork called the actin cap, through the small GTPase Ran-mediated signaling cascade that leads to Arp2/3 activation^2,3^. During Ana-II, segregated chromosomes also induce actin cap formation. The actin cap, together with the actomyosin ring that is located in the peripheral region of the actin cap, pushes the plasma membrane to form a membrane protrusion. The actomyosin-based contractile ring is then formed at the neck region of the protrusion, resulting in the formation of a polar body upon completion of cytokinesis^2,4–6^. The formation and function of the contractile ring depends on the numerous proteins accumulated at the overlapping antiparallel microtubules called the central-spindle midzone that forms between the two segregating chromatids^7^. In the anaphase, protein regulator of cytokinesis 1 (PRC1) exhibits strong microtubule bundling activity upon dephosphorylation by PP2A-B55, and plays a critical role in the spindle midzone organization^8–10^, and therefore in contractile ring assembly. During the formation of the second polar body and maternal PN, sperm-derived chromatin forms a paternal PN after extensive chromatin remodeling processes such as protamine-histone exchange^11^.

For full-term mammalian development, it is essential that the zygote is diploid with a maternal and a paternal PN. This is primarily because of the differential genomic modification, called genomic imprinting, that leads to the functional difference between paternal and maternal genomes^12–16^. Therefore, parthenogenetic and androgenetic embryos that contain only oocyte-derived or sperm-derived chromosomes are powerful and indispensable tools for the comprehensive study of maternal or paternal genome-specific modifications and functions. Parthenogenetic embryos can be obtained relatively easily by the artificial activation of Meta-II arrested oocytes, whereas the process of producing androgenetic embryos is complex. Androgenetic embryos are produced through the removal of Meta-II chromosomes and spindles from unfertilized oocytes, followed by intracytoplasmic sperm injection or *in vitro* fertilization (IVF)^12,13,16–18^. Alternatively, the maternal PN is removed from the zygote^19^. In both procedures, enucleation is accomplished by the use of a fine glass needle (injection pipette), which requires a high level of technical skill and specialized equipment. Moreover, enucleation is a time-consuming process that carries the risk of cellular damage, which can be evoked either by manipulation or the chemical treatments involved, resulting in a low oocyte survival rate.

In this study, we demonstrated that by a slight perturbation of the spindle function, whole Meta-II oocyte chromosomes could be extruded into a polar body. Spindle perturbation was then used to establish a method for producing androgenetic embryos without the invasive enucleation process.

## Results and Discussion

### Low-dose nocodazole induced the extrusion of whole oocyte chromosomes into the second polar body

In the course of studying the effect of nocodazole, a microtubule-destabilizing drug, on oocyte meiotic spindle, we prepared mineral oil-covered dishes that contained two drops of activation medium, one with 25 µg/ml nocodazole and the other without nocodazole, and added Meta-II oocytes into the nocodazole-free drop. In the nocodazole-free activation medium, we observed an oocyte with chromosomes that were not segregated but instead extruded into the second polar body, which led to the formation of an achromosomal oocyte (Video 1). Furthermore, in this oocyte, the microtubules were destabilized (Video 1). This observation implied that the nocodazole in the medium drop was diffused into the neighboring drops to some extent through the mineral oil, which have led to the formation of an achromosomal oocyte. These observations prompted us to establish the conditions for *in vitro* fertilization (IVF), under which whole oocyte chromosomes were extruded into the second polar body, resulting in the production of androgenetic embryos.

### ***In vitro*** fertilization in the presence of low-dose nocodazole produced four categories of zygotes

**Figure 1:**
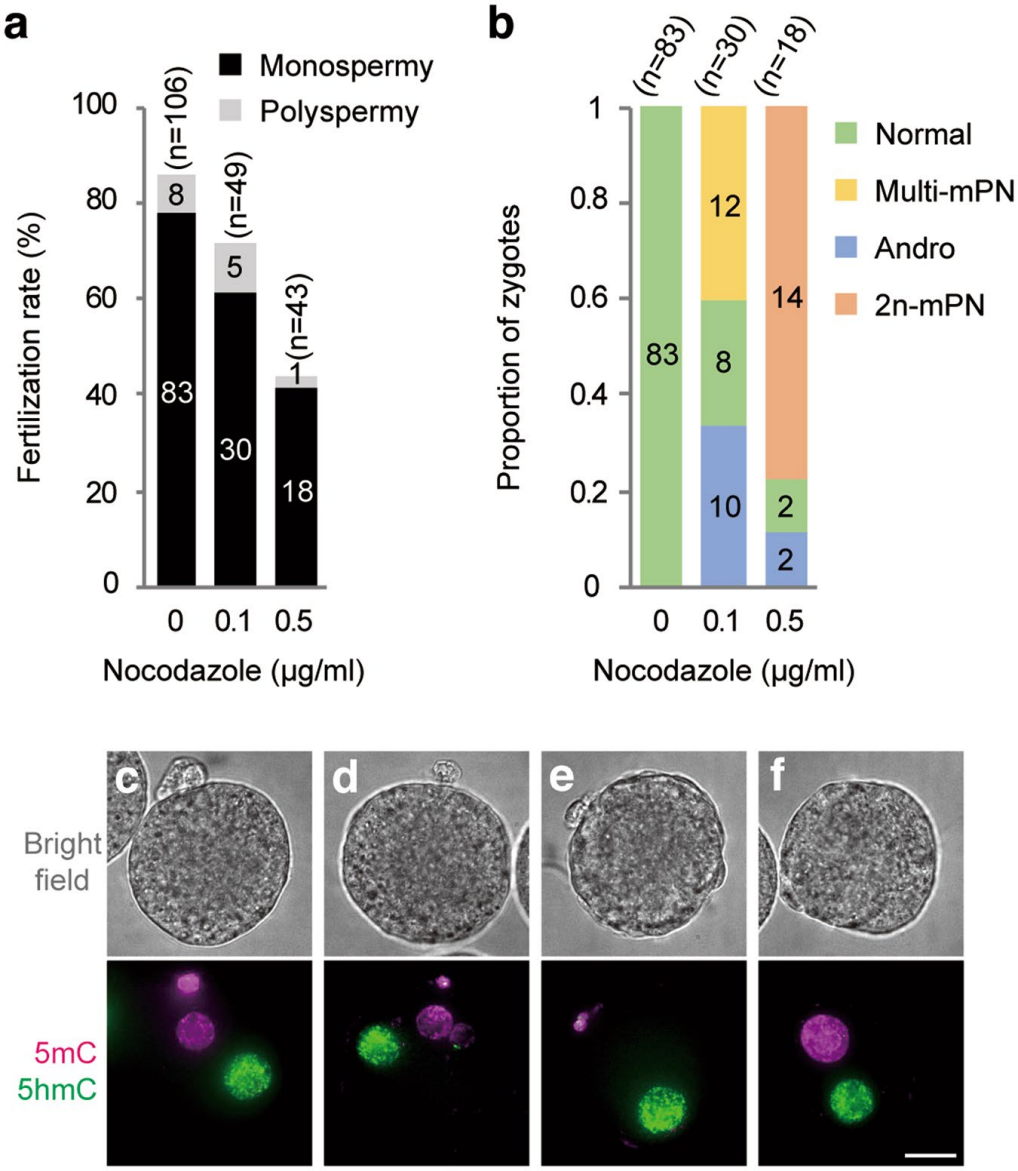
*In vitro* fertilization in the presence of low-dose nocodazole produced four categories of zygotes. (**a**) Fertilization efficiencies in the presence of low-dose nocodazole. The numbers in bars indicate the number of zygotes in each category. (**b**) The ratio of the four categories of zygotes obtained by IVF with 0, 0.1, or 0.5 µg/ml nocodazole. Normal: diploid zygote containing a paternal PN and a maternal PN. Multi-mPN: zygote containing a single paternal PN and multiple fragmented maternal PNs. Andro: haploid androgenetic embryo containing a single paternal PN. 2n-mPN: zygote containing a paternal PN and a large maternal PN that encapsulates whole oocyte chromosomes without forming a second polar body. The numbers in bars indicate the number of zygotes in each category. (**c–f**) Representative bright-field (upper panels) and immunofluorescent (lower panels) images of the zygotes from each category. Zygotes were fixed and stained for 5mC (magenta) and 5hmC (green). The numbers of oocytes used (n) are shown. Scale bar: 20 µm.

First, we performed IVF in the media containing 0, 0.1, or 0.5 µg/ml nocodazole using Meta-II oocytes obtained from ICR mice. To ensure that the degree of nocodazole diffusion was comparable between the experiments, each IVF was carried out in a separate dish containing fixed volumes of medium and mineral oil (see materials and methods section for detail). Six hours after insemination, the oocytes were fixed and stained for 5-methylcytosine (5mC) and 5-hydroxylmethylcytosine (5hmC) to examine the presence of maternal and paternal PN, respectively^20,21^. As judged by the presence of paternal PN, fertilization efficiency reached more than 70% in 0.1 µg/ml nocodazole, while 0.5 µg/ml nocodazole decreased the fertilization rate to less than 50% (Fig. 1a). Under these conditions, about 5% to 14% were polyspermic zygotes that contained two paternal PN (Fig. 1a), which were excluded from further analyses. The zygotes obtained in this series of experiments could be categorized into four groups based on the number and morphology of the maternal PN (Fig. 1b). In the absence of nocodazole, all the zygotes produced by IVF contained a larger paternal and a smaller maternal PN (hereafter called Normal) (Fig. 1b,c). In the presence of 0.1 µg/ml nocodazole, the ratio of Normal decreased to 26.7% and two other categories of zygotes were observed; zygotes that contained a single paternal PN and multiple, fragmented maternal PN (hereafter called Multi-mPN) (Fig. 1b,d), and androgenetic zygotes that contained only a single paternal PN (hereafter called Andro) (Fig. 1b,e). All zygotes formed in the presence of 0.1 µg/ml nocodazole were associated with the emission of the second polar body. These results suggest that 0.1 µg/ml nocodazole caused defects in oocyte chromosome segregation without affecting cytokinesis, leading to the formation of Multi-mPN or Andro zygotes. On the other hand, the majority of the zygotes formed in 0.5 µg/ml nocodazole failed to extrude the second polar body and contained a smaller paternal PN and a larger maternal PN that was assumed to contain whole oocyte chromosomes (hereafter called 2n-mPN) (Fig. 1b,f).

### ***In vitro*** fertilization with the optimized concentration of nocodazole produced androgenetic embryos at a high frequency

**Figure 2:**
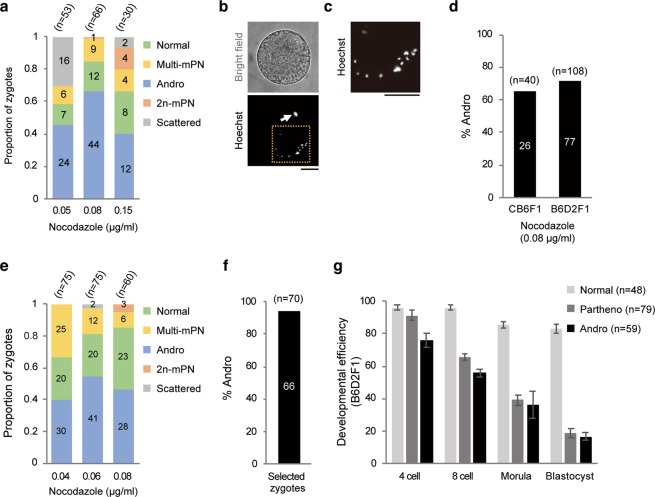
Optimization of the nocodazole concentration produced androgenetic embryos with appropriate developmental potential. (**a**) The ratio of the five categorizations of zygotes obtained by IVF with 0.05, 0.08, and 0.15 µg/ml nocodazole. The numbers in bars indicate the numbers of zygotes in each category. (**b**) Representative bright field and Hoechst-stained DNA images of the Scattered zygote. A white arrow indicates sperm-derived chromatin. (**c**) Magnified image of the dashed boxed region in (**b**). (**d**) The production efficiencies of Andro by IVF in the presence of 0.08 µg/ml nocodazole with oocytes obtained from CB6F1 and B6D2F1 mice. The numbers in bars indicate the numbers of Andro. (**e**) The ratio of the five categories of the zygotes obtained by IVF without using mineral oil. The numbers in bars indicate the numbers of zygotes in each category. (**f**) The ratio of Andro within the selected zygotes as determined by an inverted phase-contrast microscope. Oocytes obtained from B6D2F1 mice were used. The numbers in bars indicate the numbers of Andro. (**g**) Developmental efficiencies of the blastocyst of diploid zygotes (Normal), haploid parthenogenetic embryos (Partheno), and haploid androgenetic embryos produced by IVF with 0.08 µg/ml nocodazole (Andro). Oocytes obtained from B6D2F1 mice were used. Error bars represent mean ± s.e.m. (at least three independent experiments). The numbers of oocytes used (n) are shown. Scale bar: 20 µm.

We further explored the optimized conditions for the production of haploid androgenetic embryos by testing the *in vitro* fertilization with 0.05–0.15 µg/ml of nocodazole (Fig. 2a). In the presence of 0.05 µg/ml nocodazole, Multi-mPN and Andro as well as zygotes with a different abnormality were produced by IVF. More than 20% of zygotes contained scattered, meiotically condensed oocyte chromosomes and a slightly swelled sperm nuclei (hereafter called Scattered zygotes) (Fig. 2a–c). Even though sperm-oocyte fusion occurred, Scattered zygotes remained in the meiotic phase for unknown reasons. The formation of Andro was the most efficient in the presence of 0.08 µg/ml nocodazole (Fig. 2a). When the Meta-II oocytes obtained from CB6F1 or B6D2F1 female mice were used for IVF in the presence of 0.08 µg/ml of nocodazole, 65% and 71.3% of zygotes became Andro, respectively (Fig. 2d), implying that this was not an ICR-specific phenomenon. Moreover, to prevent nocodazole from diffusing from the medium and to demonstrate the reproducibility of our method, we performed IVF experiments in a well with 350 µl of nocodazole-containing medium not covered by mineral oil. Expectedly, the formation of Andro was the most efficient in the presence of 0.06 µg/ml nocodazole, which is a concentration lower than what is optimally recommended for IVF with mineral oil (Fig. 2e).

Next, we examined the developmental efficiency of the haploid androgenetic embryos obtained by the nocodazole-induced method. We observed the obtained zygotes using an inverted phase-contrast microscope and selected zygotes with a second polar body and a single PN. Immunofluorescent staining revealed that more than 94% of the selected zygotes were indeed Andro (Fig. 2f), and the remaining zygotes were multi-mPN. On day 5, 16.7% of the selected Andro developed into blastocysts *in vitro*. The developmental efficiency of Andro was comparable with that of haploid parthenogenetic embryos (Fig. 2g, Supplementary Material Fig. S1) and was consistent with the previously reported results^16,22^.

### An intrinsic cytokinetic mechanism was used to exclude maternal chromatin from the zygotic genome

**Figure 3:**
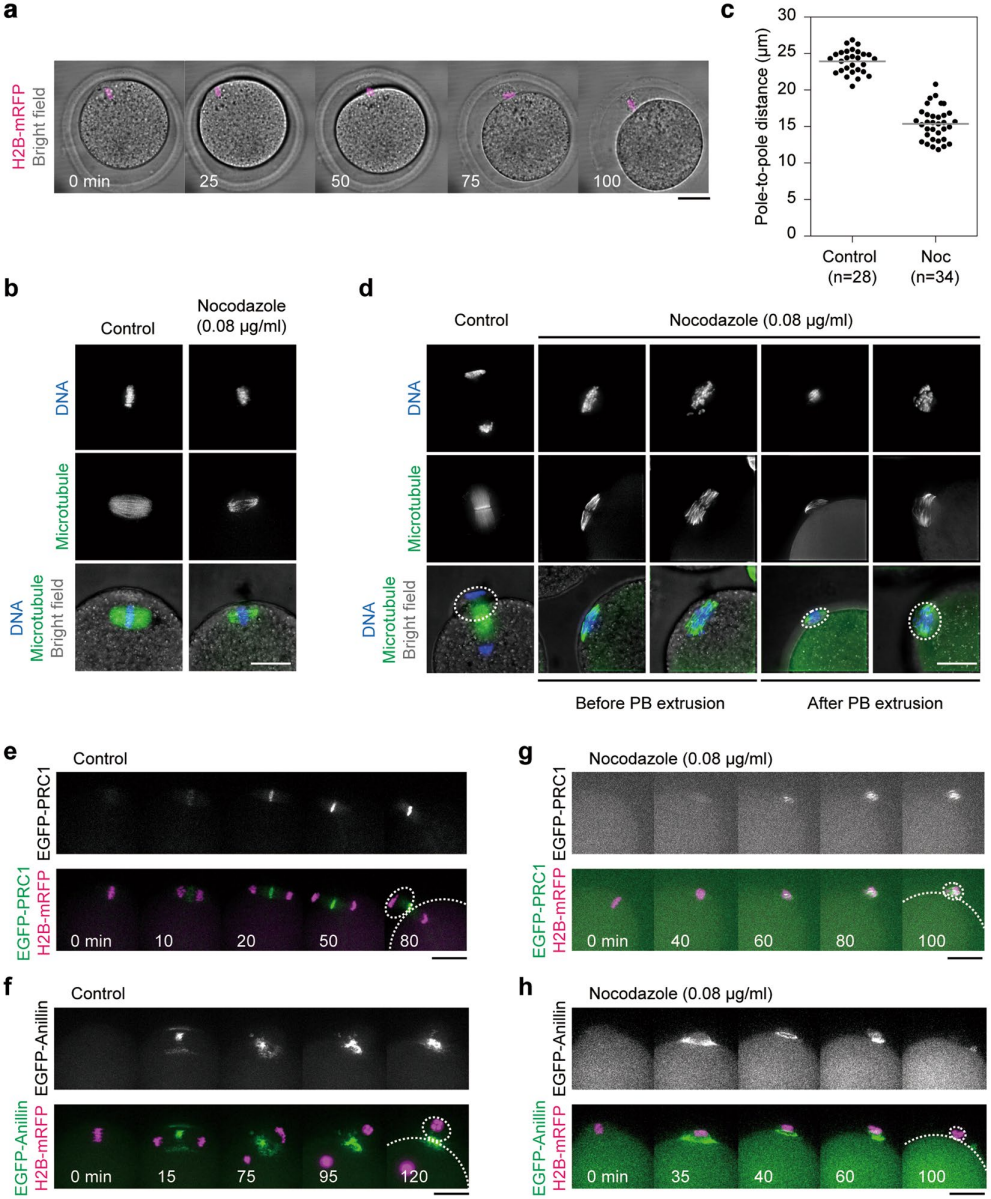
Small spindles with unsegregated chromosomes induced protrusion formation and contractile ring assembly. (**a**) The formation of achromosomal oocytes by Sr^2+^-mediated activation with 0.08 µg/ml nocodazole. An oocyte expressing H2B-mRFP (merged with bright-field) was subjected to live imaging. The numbers indicate the time after activation (min). **(b**) Representative images of Meta-II spindle in the presence or absence of 0.08 µg/ml nocodazole. Meta-II oocytes were cultured for 30 min with (Nocodazole) or without (Control) nocodazole, fixed, and stained for microtubules (green) and DNA (blue). (**c**) Pole-to-pole distance of the Meta-II spindle before (Control) or after 30 min treatment with 0.08 µg/ml nocodazole (Noc). Bars indicate average values of each category. (**d**) Representative images of parthenogenetically activated oocytes with (Nocodazole) or without (Control) 0.08 µg/ml nocodazole. Oocytes were fixed 65 min after activation and stained for microtubules (green) and DNA (blue). The second polar body is outlined by the white dashed line in the lowest panels. (**e**) Dynamics of EGFP-PRC1 during the second polar body formation. Oocytes expressing EGFP-PRC1 and Histone H2B-mRFP1 were activated and subjected to live imaging. The numbers in the lower panels indicate the time after Ana-II onset (min). (**f**) Dynamics of EGFP-Anillin during the second polar body formation. Oocytes expressing EGFP-Anillin and Histone H2B-mRFP1 were analyzed as in (**e**). (**g**) Dynamics of EGFP-PRC1 in the activated oocytes in the presence of 0.08 µg/ml nocodazole. Oocytes expressing EGFP-PRC1 and Histone H2B-mRFP1 were activated in the presence of nocodazole and subjected to live imaging. The numbers in the lower panels indicate the time after activation (min). (**h**) Dynamics of EGFP-Anillin during the second polar body formation. Oocytes expressing EGFP-Anillin and Histone H2B-mRFP1 were analyzed as in (**g**). Scale bar: 20 µm.

To investigate the impact of nocodazole on chromosome dynamics, Meta-II oocytes expressing mRFP1-tagged Histone H2B were cultured in a drop of activation medium with 0.08 µg/ml nocodazole and subjected to live imaging. As in the first observation, chromosomes were not segregated but instead moved closer to the plasma membrane, which led to the formation of a protrusion that eventually became a polar body that contained all the oocyte chromosomes (Fig. 3a and Video 2). Immunofluorescent staining revealed that in the presence of 0.08 µg/ml nocodazole, the average pole-to-pole distance of the Meta-II spindle was reduced from 23.9 ± 1.57 to 15.4 ± 2.20 µm within 30 minutes (Fig. 3b,c). We confirmed that reduction in spindle size was recovered by removing nocodazole from the medium (Supplementary Material Fig. S2), suggesting that the effect of the low-dose nocodazole was reversible. At 65 min after activation in the sample without nocodazole, the chromosomes were segregated, and the polar body was already formed in the majority of the oocytes (Fig. 3d). In contrast, consistent with the live-image observation, chromosomes remained unsegregated on the small spindle in the presence of 0.08 µg/ml nocodazole. It appears that because of the reduction in the size of the spindle, the chromosomes were located close to the cortex possibly by Arp2/3-dependent cytoplasmic streaming^23^. After the formation of the second polar body, the zygote and the polar body remained connected by the bundle of the microtubules in the absence of nocodazole. In the presence of 0.08 µg/ml nocodazole, however, microtubules were only observed in the polar body (Fig. 3d). Presumably for this reason, the polar body was often detached from the Andro zygote during preparation for immunofluorescent staining.

**Figure 4:**
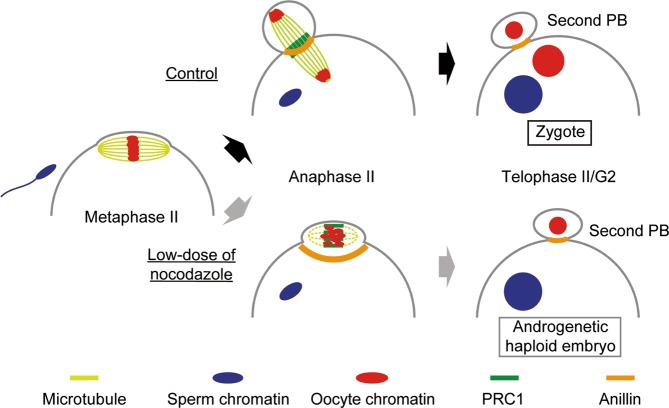
Schematic model for the nocodazole-induced haploid androgenetic embryo formation. Upon fusion with sperm, oocytes enter Ana-II. Low-dose nocodazole decreases the size and microtubule density of the spindle and prevents chromosome segregation. As a result, unsegregated chromosomes move closer to the plasma membrane and induce the formation of a protrusion. Although the central spindle is not formed, PRC1 accumulates on the microtubules around the chromosomes and triggers the formation of a contractile ring around the neck region of the protrusion, leading to the formation of the second polar body that contains all of the oocyte chromosomes.

To examine the localization of the proteins that are responsible for the contractile ring formation, we observed the dynamics of PRC1 and Anillin. In somatic cells, Anillin, a conserved component of the contractile ring, is recruited to the cell cortex close to the central spindle midzone, whose formation is dependent on PRC1 accumulation on the central spindle^24–26^. In mouse oocytes, Anillin has been reported to be essential for the extrusion of polar bodies^27^. In control oocytes, EGFP-PRC1 accumulated at the central spindle about 10 min after the onset of Ana-II and was localized at the midzone (Fig. 3e and Video 3). EGFP-Anillin signals at the plasma membrane in the vicinity of the midzone became prominent 15 min after Ana-II onset, and a ring-like structure was observed when the second polar body was formed (Fig. 3f and Video 4). The data were consistent with the localization pattern of PRC1 and Anillin observed in Ana-II oocytes by immunostaining^28,29^. In the presence of 0.08 µg/ml nocodazole, despite the absence of chromosome segregation, weak but significant accumulation of EGFP-PRC1 in a stripe-like pattern was observed near the chromosomes and was maintained even after the formation of a polar body (Fig. 3g and Video 5). Strikingly, EGFP-Anillin formed a ring-like structure, not around the spindle equator, but at the neck region of the protrusion where the ring shrunk as cytokinesis proceeded (Fig. 3h and Video 6). Taken together, we propose the following model for nocodazole-induced androgenetic embryo formation (Fig. 4). Low-doses, most efficiently 0.08 µg/ml nocodazole, did not affect sperm-oocyte fusion but decreased the size as well as the microtubule density of the spindle and often prevented chromosome segregation without reactivating the spindle assembly checkpoint. As a result, the cytoplasm of the oocyte proceeded to the anaphase state that induced the formation of a protrusion around the chromosomes and accumulation of PRC1 on the microtubules. Accumulated PRC1 then triggered the formation of a contractile ring around the neck region of the protrusion, which led to the formation of the second polar body that contained all of the oocyte chromosomes.

In summary, we developed a novel method for producing mouse androgenetic embryos. In this method, instead of glass needle-mediated micromanipulations, an intrinsic cytokinetic mechanism is used to exclude maternal chromatin from the zygotic genome. Therefore, our method is free from the damage associated with conventional and invasive procedures^30,31^. Moreover, our method does not require a high level of technical skill for enucleation or expensive micromanipulation systems, and a large number of androgenetic embryos can be obtained simultaneously. During the process of conversion of sperm nuclei to paternal pronuclei, sperm chromatin undergoes several dynamic changes, including protamine removal and histone incorporation that eventually result in differences in epigenetic modifications compared to maternal pronuclei^32–36^. This process requires a longer time in mammals than in non-mammalian vertebrates^37,38^ and raises intriguing questions regarding the molecular events that must be completed during this process and the exact mechanisms underlying asymmetrical regulation between paternal and maternal chromatins. Our method will allow a wide range of researchers, who do not have experience with enucleation techniques, to address these issues. In addition, our method may be applicable to many other mammals by determining the appropriate concentration of nocodazole or other microtubule drugs. With these advantages, our developed method can contribute to a deeper understanding of mammalian development.

## Materials and methods

### Oocyte and sperm collection

ICR and B6D2F1 mice were purchased from Charles River Laboratories. To obtain Balb/cA x C57BL/6J F1 (CB6F1) mice, female Balb/cA and male C57BL/6J were purchased from CLEA Japan and crossed-bred. Female mice (8–12 weeks old) were superovulated with 5 IU pregnant mare serum gonadotropin (ZENOAQ) for 48 h, and 5 IU human chronic gonadotropin (Kyoritsu seiyaku) for 18–20 h prior to oocyte collection. Unless otherwise noted, oocyte-cumulus complexes were collected in M2 medium (M7167, Sigma-Aldrich) containing 100 µg/mL hyaluronidase, denuded from cumulus cells and cultured in M16 medium (M7292, Sigma-Aldrich) covered with mineral oil (M8410, Sigma-Aldrich) at 37 °C under 5% CO_2_ until mRNA injection or parthenogenic activation. Sperm were collected from male CB6F1 (8–12 weeks old) and precultured in 80 µl HTF medium (ARK Resource) covered with mineral oil for 1 h at 37 °C under 5% CO_2_. The experiments with animals were approved by the Animal Experimentation Committee of Graduate School of Arts and Sciences, The University of Tokyo (approval no. 26–29) and performed in accordance with the guidelines for animal use issued by the Committee of Animal Experiments, The University of Tokyo.

### Parthenogenetic activation

Parthenogenetic activation was carried out using the strontium-induced method^39^. Meta-II arrested oocytes were incubated in activation medium (M16 medium supplemented with 5 mM SrCl_2_ and 5 mM EGTA) with or without nocodazole and cultured at 37 °C under 5% CO_2_. To examine the developmental potential of haploid parthenogenetic embryos, oocytes were transferred into M16 medium three hours after activation.

### *In vitro* fertilization (IVF)

Oocyte-cumulus complexes from superovulated female mice were collected and placed in 50 µl of M16 medium with or without nocodazole. Sperms separated using the swim-up method were added so that the final concentration of sperm was 2.0 ×10^6^/ml and was then cultured at 37 °C under 5% CO_2_. To minimize the effect of nocodazole on zygotic development so that the developmental potential of haploid androgenetic embryos could be determined, oocytes were transferred into M16 medium 3 h after insemination in the experiments shown in Fig. 2f,g. When IVF was performed without using mineral oil, oocyte-cumulus complexes were transferred to a well in a 48-well plate containing 350 µl of M16 medium with nocodazole and subjected to insemination. To minimize the effect of medium evaporation on zygotes, 3 h post-insemination, oocytes were transferred from a 48-well plate into an M16 medium drop covered with mineral oil.

### Preparation of the nocodazole-containing medium

Nocodazole (M1404, Sigma-Aldrich) was dissolved in dimethyl sulfoxide (DMSO) (Wako) at a concentration of 5 mg/ml, dispensed into small aliquots, and stored at −20 °C until use. For the preparation of the nocodazole containing medium, nocodazole was diluted in two steps. Freshly thawed nocodazole was first diluted with DMSO and then diluted with M16 medium or activation medium. M16 medium and activation medium used in the second dilution step was pre-incubated at 37 °C under 5% CO_2_ for 24 h before use. The dilution ratio of the first step was determined so that the dilution ratio of the second step was always 200-fold to achieve the final concentration. Re-frozen nocodazole was not used in any of the experiments. In order to keep the amount of nocodazole that diffuses into mineral oil constant in all experiments, the volume of nocodazole-containing medium drop and the volume of mineral oil, as well as the time from the preparation of the medium drop covered with oil to the start of insemination or activation were kept constant as follows. A 50 µl medium drop or a 3 µl and a 47 µl drop for live imaging, was placed on a φ 3.5 cm dish and covered with 4 ml mineral oil and incubated at 37 °C under 5% CO_2_ for 70 to 80 min before insemination or parthenogenetic activation. When using a 48-well plate, 350 µl of nocodazole-containing medium was placed in a well and incubated at 37 °C under 5% CO_2_ for 70 to 80 min before insemination.

### *In vitro* development of zygotes and parthenogenetic embryos

Six hours after insemination or activation, oocytes were observed under the inverted phase-contrast microscope (IX71; OLYMPUS) equipped with a 40×/0.6 RC3 objective lens (OLYMPUS). Only those that extruded the second polar body and formed two (for Normal zygotes) or one (for haploid parthenogenetic and androgenetic embryos) PN were selected and subjected to further culture for 5 days at 37 °C under 5% CO_2_.

### mRNA preparation and microinjection

mRNAs were synthesized *in vitro* with linearized template plasmids using a RiboMax Large Scale RNA Production System-T7 (Promega) supplemented with Ribo m^7^G Cap Analog (Promega) in reaction mixtures as described previously^40^. Template plasmids for EGFP-Anillin-Cterminal were constructed using cDNA from a pEGFP-Anillin-Cterminal vector^41^. Template plasmids for EGFP-PRC1 were constructed using human full-length PRC1 cDNA PCR amplified from the HeLa cDNA library. Template plasmids encoding Histone H2B-mRFP1 and EGFP-alpha-tubulin were described previously^42^. A few picoliters of mRNA (10–200 ng/µl) were microinjected into Meta-II oocytes with a Piezo-driven micromanipulator (Prime Tech) and cultured for 4 h before being subjected to parthenogenetic activation and live-cell imaging.

### Live-cell imaging

mRNA-injected oocytes were placed into 3 µl drops of activation medium with or without nocodazole and covered with mineral oil in a glass-bottom dish (MatTek), which was then placed in a stage top incubator at 37 °C under 5% CO_2_. Confocal images were collected with a microscope (IX71; OLYMPUS) equipped with a spinning disk confocal system (CSU10; Yokogawa), 60×/1.30 Sil objective lens (OLYMPUS), and CCD camera (iXon DU897E-CSO-#BV; ANDOR) controlled by Metamorph software (Universal Imaging). Confocal images were collected as z-stacks at 1 µm intervals (number of optical z-stacks: 16) to visualize the entire spindle.

### Nocodazole removal assay

After 30 min incubation in 50 µl of M16 medium containing 0.08 µg/ml nocodazole, Meta-II oocytes were washed thrice with 30 µl of nocodazole-free M16 medium and then cultured in 50 µl of nocodazole-free M16 medium for 3 h before fixation.

### Immunofluorescent staining

After the removal of the zona pellucida with acidic Tyrode’s solution, oocytes were washed with 0.5% polyvinyl pyrrolidone/PBS, fixed in 4% paraformaldehyde for 30 min at 25 °C, permeabilized with 0.2% Triton-X100/PBS for 30 min at 25 °C, and placed in blocking solution (PBS containing 3% BSA and 0.1% Tween20) overnight at 4 °C. For 5-hydroxymethylcytosine (5hmC) and 5-methylcytosine (5mC) staining, permeabilization was carried out with 2 N HCl and 0.5% Triton-X100/PBS. After blocking, samples were incubated for 1 h with primary antibody solutions, washed three times for 20 min, incubated for 1 h with secondary antibody solutions supplemented with Hoechst 33342 dye (10 µg/ml, H-1399, Thermo Fisher Scientific) to visualize chromosomes, and washed three times for 20 min. The primary antibodies used in this study were rabbit anti-5hmC (1:1000, 39769, Active Motif), mouse anti-5mC (1:1000, D346-3, MBL), and rat anti-α-tubulin (1:1000, sc-53029, Santa Cruz Biotechnology, YL1/2). Secondary antibodies were Alexa Fluor 488-conjugated goat anti-rat (1:500, A-11006, Invitrogen), goat anti-rabbit (1:500, A-11034, Invitrogen), or Alexa Fluor 555-conjugated goat anti-mouse (1:500, A-21424, Thermo Fisher Scientific). Fluorescent images were collected as z-stacks at 2 µm intervals (number of optical z-stacks: 45) to visualize entire zygotes, using a fluorescence microscope (IX70; OLYMPUS) equipped with a 60×/1.30 NA Sil objective lens (OLYMPUS), and a cold CCD camera (CoolSNAP HQ; Roper Scientific) that was controlled using Delta Vision SoftWorx (Applied Precision). Image stacks were deconvoluted, quick-projected, and analyzed with ImageJ software.

## Supplementary information

Supplementary Information.

Supplementary Video 1.

Supplementary Video 2.

Supplementary Video 3.

Supplementary Video 4.

Supplementary Video 5.

Supplementary Video 6.
