# Supplementary material for: Production of mouse androgenetic embryos using spindle perturbation
Source: Sci Rep. 2020 Apr 16;10:6556. doi: 10.1038/s41598-020-63010-x (PMC7162913; doi:10.1038/s41598-020-63010-x)
Supplement: Supplementary file 1 — Supplementary Information. [file 41598_2020_63010_MOESM1_ESM.pdf]

Title

Production of mouse androgenetic embryos using spindle perturbation

Authors

Takaya Totsuka[1,2] and Miho Ohsugi[1,2] *

*Author for correspondence (mohsugi@bio.c.u-tokyo.ac.jp)

Affiliations

1. Department of Biological Sciences, Graduate School of Science, The University of Tokyo, Hongo 7-3-1, Bunkyo-ku, Tokyo 113-0033, Japan.

2. Department of Life Sciences, Graduate School of Arts and Sciences, The University of Tokyo, Komaba 3-8-1, Meguro-ku, Tokyo 153-8902, Japan.

Supplementary information

Video 1. Formation of the second polar body under the influence of nocodazole.
Time-lapse observation of oocytes expressing Histone H2B-mRFP1 (magenta) and EGFP-alpha-tubulin (green) in the activation medium drop, with (right) or without (left) a nocodazole-containing medium drop in the same oil-covered dish.

Video 2. Formation of achrosomal oocytes in the presence of 0.08 μg/ml nocodazole.
Time-lapse observation of oocytes expressing Histone H2B-mRFP1 (magenta) in the activation medium containing 0.08 μg/ml nocodazole.

Video 3. Accumulation of EGFP-PRC1 to the midzone in control Ana-II oocytes.
Time-lapse observation of oocytes expressing Histone H2B-mRFP1 (magenta) and EGFP-PRC1 (green) in the activation medium.

Video 4. Formation of a contractile ring around the midzone in the control Ana-II oocytes.
Time-lapse observation of oocytes expressing Histone H2B-mRFP1 (magenta) and EGFP-Anillin (green) in the activation medium.

Video 5. Accumulation of EGFP-PRC1 around unsegregated chromosomes in nocodazole treated Ana-II oocytes.
Time-lapse observation of oocytes expressing Histone H2B-mRFP1 (magenta) and EGFP-PRC1 (green) in the activation medium containing 0.08 μg/ml nocodazole.

Video 6. Formation of the contractile ring around the neck region of the protrusion in nocodazole treated Ana-II oocytes.
Time-lapse observation of oocytes expressing Histone H2B-mRFP1 (magenta) and EGFP-Anillin (green) in the activation medium containing 0.08 μg/ml nocodazole.

Figure S1. Embryos obtained by 5-day in vitro culture after IVF or activation.
Images of embryos developed from zygotes obtained by IVF (a), haploid parthenogenetic embryos obtained by parthenogenetic activation (b), and haploid androgenetic embryos obtained by IVF in the presence of 0.08 μg/ml nocodazole (c).

Figure S2. Reversibility of the effect of the low-dose nocodazole.

(a) Representative images of the Meta-II spindle before, after and washout of nocodazole treatment. Oocytes were fixed and stained for microtubules (green) and DNA (blue) before (Control) or after 30 min incubation with 0.08 µg/ml nocodazole (Nocodazole), or after an additional 3 h incubation in nocodazole-free medium after nocodazole treatment (Washout). Quantification of Meta-II spindle size after removing the nocodazole. The numbers of oocytes used (n) are shown. Scale bar : 20 µm.

Supplementary Information

Supplementary figures

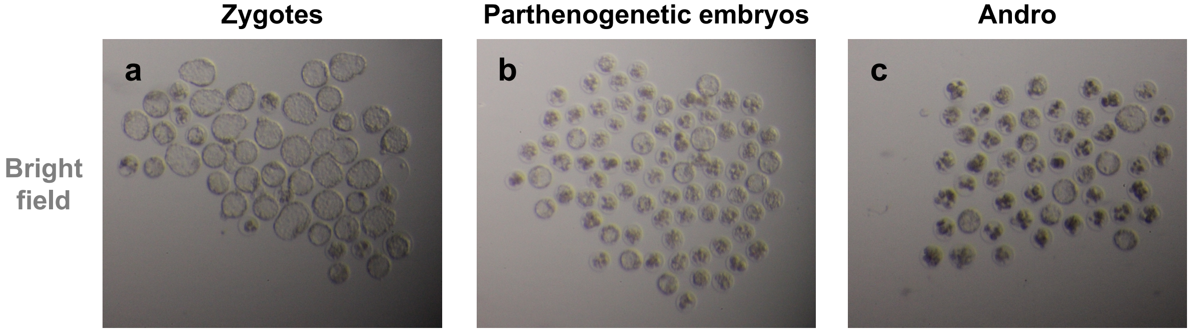

Figure S1. Embryos obtained by 5-day in vitro culture after IVF or activation.
Images of embryos developed from zygotes obtained by IVF (a), haploid parthenogenetic embryos obtained by parthenogenetic activation (b), and haploid androgenetic embryos obtained by IVF in the presence of 0.08 µg/ml nocodazole (c).

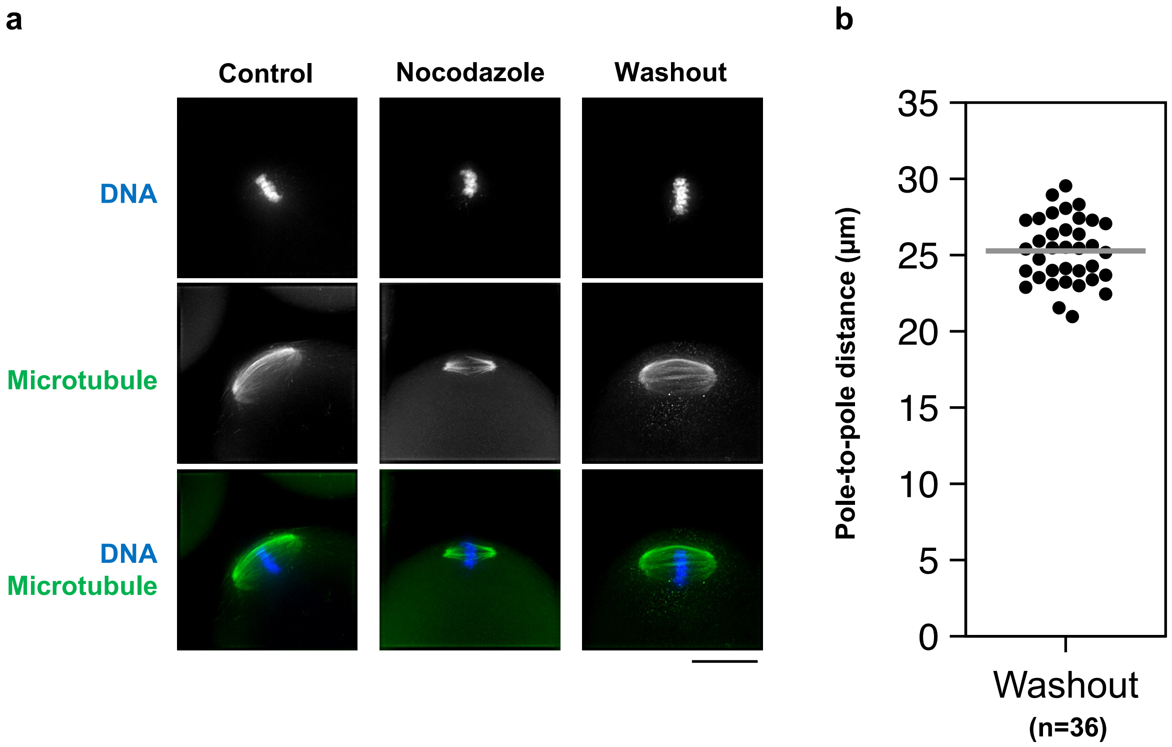

Figure S2. Reversibility of the effect of the low-dose nocodazole.

(a) Representative images of the Meta-II spindle before, after and washout of nocodazole treatment. Oocytes were fixed and stained for microtubules (green) and DNA (blue) before (Control) or after 30 min incubation with 0.08 µg/ml nocodazole (Nocodazole), or after an additional 3 h incubation in nocodazole-free medium after nocodazole treatment (Washout). Quantification of Meta-II spindle size after removing the nocodazole. The numbers of oocytes used (n) are shown. bar : 20 µm.
